# Correction to: ‘The natural selection of bad science’ (2016) by Paul E. Smaldino and Richard McElreath

**DOI:** 10.1098/rsos.231026

**Published:** 2023-09-06

**Authors:** Paul E. Smaldino, Richard McElreath

*R. Soc. Open Sci*. **3**, 160384. (Published online 1 September 2016) (doi:10.1098/rsos.160384).

Owing to a coding error in the implemented Java model, laboratories would always use the initial replication rate as probability to conduct replications, instead of their own replication rate. In particular, in the function definition of chooseHypothesis(AgentsSimulation) inside the file Boffin.java, lines 86–102, the initial replication rate as.initReplication has been used, but instead the laboratory's own property replicationProb should have been used. Hence, the replication rate could not influence the laboratories' fitness, although it could mutate. As a result, the resulting dynamics presented in §5.2 and depicted in figure 5 do not correspond to the conceptual model described in §4.

The implemented Java model also reverses the order in which laboratories are removed from the population and chosen to reproduce during the stage *evolution*, which is a minor deviation from the description in §4.

Finally, §5.3 contains two typos concerning high and low efforts. The correct values are *e*_H_ = 75 and *e*_L_ = 15.

The paper's conclusions remain unaffected after correcting for these errors. For further discussion, see [1], which also presents a reimplementation of the model in the R programming language, archived in the Software Heritage archive at https://archive.softwareheritage.org/swh:1:snp:60ab9f391840fbb0d226fdbce35169b271e00918;origin=https://gitlab.com/fkohrt/bachelorarbeit-code.
