# Supplementary material for: The natural selection of bad science
Source: R Soc Open Sci. 2016 Sep 21;3(9):160384. doi: 10.1098/rsos.160384 (PMC5043322; doi:10.1098/rsos.160384)
Supplement: 1. Methods for Meta-Analysis of Statistical Power [file rsos160384supp1.pdf]

SI TEXT
THE NATURAL SELECTION OF BAD SCIENCE

PAUL E. SMALDINO[1] AND RICHARD MCELREATH[2,3]

Methods for Meta-Analysis of Statistical Power

The first review of statistical power in published research was performed by Cohen (1962). Two decades later, two retrospective analyses were performed by Sedlmeier and Gigerenzer (1989) and Rossi (1990) to examine the possibility of any changes in the statistical power of published research since Cohen's original study. Neither paper found any change.

We built on this research by aggregating reviews and meta-analyses from the social and behavioral sciences that looked at the statistical power in the published literature. To do this, we searched Google Scholar for publications citing Sedlmeier and Gigerenzer's 1989 paper (by far the more heavily cited of the two original reviews), using the search terms "statistical power" and "review." We selected only those papers that contained reviews of statistical power from published papers in the social and behavioral sciences, and specifically estimated power for small effect sizes ($d = 0.2$ or equivalent). We restricted ourselves to small effects because, due to the large number of externalities in social and behavioral research, many effects should in fact be small. This led us to discard three papers that otherwise measured statistical power. The authors of the studies used all implemented Cohen's (1992) methods for the calculation of statistical power, assuming a rate of Type I errors of $\alpha = 0.05$. Our search yielded 16 additional review papers published between 1992 and 2014. One of these papers, Ison (2011), separately reported power for four different journals using widely different ranges of publication years. We therefore separately report these as four distinct data points. For all other reviews, some reported on single journals, while others used several journals within a given discipline. Some of these reports were for all papers published within a single year, while others reported the average power for papers published within a range of years. In the latter case, we report these data using the median year in the range. Importantly, we report data in terms of the year the original research papers, rather than the review, were published. The data for Figure 1 in the main text, including the sources, are given in Table 1.

[1]Department of Political Science, University of California-Davis, Davis, CA 95616
[2]Department of Anthropology, University of California-Davis, Davis,CA 95616
[3]Max Planck Institute for Evolutionary Anthropology, Leipzig, Germany
E-mail address: paul.smaldino@gmail.com.

Year	Effect Size			Field	Reference
	Small	Medium	Large		
1960	0.18	0.48	0.83	abnormal & social psych.	Cohen 1962[a,b]
1970	0.14	0.58	0.78	education	Brewer 1972[a,b]
1970	0.15	0.54	0.83	education	Jones & Brewer 1972[b]
1970	0.22	0.71	0.87	education	Pennick & Brewer 1973[b]
1970	0.21	0.72	0.96	education	Brewer & Owen 1973[a,b]
1970	0.19	0.46	0.72	education	Haase 1974[a,b]
1971	0.23	0.56	0.79	communication	Katzer & Sodt 1973[a,b]
1972	0.55	0.84	0.94	sociology	Spreitzer & Chase 1974[b]
1973	0.18	0.52	0.79	communication	Chase & Tucker 1975[a,b]
1973	0.16	0.44	0.73	communication	Kroll & Chase 1975[a,b]
1974	0.34	0.76	0.91	communication	Chase & Baran 1976[a,b]
1974	0.25	0.67	0.86	applied psychology	Chase & Chase 1976[a,b]
1975	0.18	0.39	0.62	education	Christensen & Christensen 1977[b]
1976	0.38	0.62	0.81	physical anthropology	Chase et al. 1978[b]
1978	0.23	0.63	0.85	education	Woolley & Dawson 1983[b]
1979	0.41	0.89	0.98	marketing	Sawyer & Ball 1981[a,b]
1979	0.22	0.63	0.86	education	Daly & Hexamer 1983[b]
1980	0.37	0.65	0.93	occupational therapy	Ottenbacher 1982[b]
1981	0.23	0.69	0.90	education	Wooley 1983[b]
1981	0.31	0.76	0.92	social work	Orme & Combs-Orme 1986[b]
1981	0.20	0.68	0.88	social work	Orme & Tolman 1986[b]
1982	0.17	0.57	0.83	abnormal & social psych.	Rossi 1990
1983	0.09	0.51	0.92	clinical psychology	Acklin et al. 1992
1984	0.12	0.37	0.86	abnormal & social psych.	Sedlmeier & Gigerenzer 1989[b]
1984	0.23	0.59	0.83	management	Marzen et al. 1987a[b]
1984	0.31	0.77	0.91	management	Marzen et al. 1987b[b]
1989	0.09	0.37	0.76	psychotherapy	Kazantis 2000
1989	0.13	0.64	0.97	music education	Daniel 1993
1990	0.10	0.52	0.92	psychology education	Tomcho & Foels 2009
1993	0.49	0.65	0.85	operations research	Verma & Goodale 1995
1993	0.27	0.74	0.92	management	Mone et al. 1996
1995	0.29	NA	NA	management	Cashen & Geiger 2004
1995	0.23	0.71	0.93	behavioral accounting	Borkowski et al. 2001
1996	0.14	0.41	0.62	aviation	Ison 2011
1997	0.36	0.77	0.92	health psychology	Maddock & Rossi 2001
1998	0.16	0.70	0.92	aviation	Ison 2011
1999	0.11	0.36	0.63	software engineering	Dyba et al. 2006
2000	0.15	0.43	0.69	behavioral ecology	Jennions & Møller 2003
2000	0.06	0.13	0.25	neuroscience	Woods et al. 2006
2000	0.43	0.75	0.91	aviation	Ison 2011
2005	0.45	0.99	1.00	international business	Zhan 2013
2005	0.27	0.61	0.80	aviation	Ison 2011
2008	0.17	NA	NA	social & personality psych.	Fraley & Vazire 2014
2011	0.27	0.54	0.95	neuroscience	Button et al. 2013

TABLE 1. Power estimates based on the median year of papers published in each reference. [a]Included in Sedlmeier & Gigerenzer (1989). [b]Included in Rossi (1990). NA indicates that power calculations were not reported in the original review papers.

References

Acklin, M. W., McDowell II, C. J., and Orndoff, S. (1992). Statistical power and the rorschach: 1975-1991. *Journal of Personality Assessment*, 59(2):366–379.

Borkowski, S. C., Welsh, M. J., and Zhang, Q. (2001). An analysis of statistical power in behavioral accounting research. *Behavioral Research in Accounting*, 13(1):63–84.

Button, K. S., Ioannidis, J. P., Mokrysz, C., Nosek, B. A., Flint, J., Robinson, E. S., and Munafò, M. R. (2013). Power failure: Why small sample size undermines the reliability of neuroscience. *Nature Reviews Neuroscience*, 14(5):365–376.

Cashen, L. H. and Geiger, S. W. (2004). Statistical power and the testing of null hypotheses: A review of contemporary management research and recommendations for future studies. *Organizational Research Methods*, 7(2):151–167.

Cohen, J. (1962). The statistical power of abnormal-social psychological research: A review. *Journal of Abnormal and Social Psychology*, 65(3):145–153.

Cohen, J. (1992). Statistical power analysis. *Current Directions in Psychological Science*, 1:98–101.

Daniel, T. D. (1993). *A Statistical Power Analysis of the Quantitative Techniques Used in the "Journal of Research in Music Education," 1987 through 1991*. PhD thesis, Auburn University, Birmingham, AL.

Dybå, T., Kampenes, V. B., and Sjøberg, D. I. (2006). A systematic review of statistical power in software engineering experiments. *Information and Software Technology*, 48(8):745–755.

Fraley, R. C. and Vazire, S. (2014). The n-pact factor: Evaluating the quality of empirical journals with respect to sample size and statistical power. *PLoS ONE*, 9(10):e109019.

Ison, D. C. (2011). An analysis of statistical power in aviation research. *International Journal of Applied Aviation Studies*, 11(1):67.

Jennions, M. D. and Møller, A. P. (2003). A survey of the statistical power of research in behavioral ecology and animal behavior. *Behavioral Ecology*, 14(3):438–445.

Kazantzis, N. (2000). Power to detect homework effects in psychotherapy outcome research. *Journal of Consulting and Clinical Psychology*, 68(1):166.

Maddock, J. E. and Rossi, J. S. (2001). Statistical power of articles published in three health-psychology related journals. *Health Psychology*, 20(1):76.

Mone, M. A., Mueller, G. C., and Mauland, W. (1996). The perceptions and usage of statistical power in applied psychology and management research. *Personnel Psychology*, 49(1):103–120.

Rossi, J. S. (1990). Statistical power of psychological research: What have we gained in 20 years? *Journal of Consulting and Clinical Psychology*, 58(5):646.

Sedlmeier, P. and Gigerenzer, G. (1989). Do studies of statistical power have an effect on the power of studies? *Psychological Bulletin*, 105(2):309–316.

Tomcho, T. J. and Foels, R. (2009). The power of teaching activities: Statistical and methodological recommendations. *Teaching of Psychology*, 36(2):96–101.

Verma, R. and Goodale, J. C. (1995). Statistical power in operations management research. *Journal of Operations Management*, 13(2):139–152.

Woods, S. P., Rippeth, J. D., Conover, E., Carey, C. L., Parsons, T. D., and Tröster, A. I. (2006). Statistical power of studies examining the cognitive effects of subthalamic nucleus deep brain stimulation in parkinson's disease. *The Clinical Neuropsychologist*, 20(1):27–38.

72 Zhan, G. (2013). Statistical power in international business research: Study levels and data types. *International Business Review*, 22(4):678–686.
